# California Pharmacists’ Perspectives and Experiences with Prayer and Spiritual Conversations with Patients: An Exploratory Study

**DOI:** 10.1007/s10943-025-02269-8

**Published:** 2025-02-12

**Authors:** Paul Gavaza, Soryal Kyrillos, Bhaktidevi Rawal, Elizabeth Johnston Taylor

**Affiliations:** 1 Loma Linda University School of Pharmacy, 24745 Stewart Street, Loma Linda, CA 92350 USA; 2 Loma Linda University School of Nursing, 11262 Campus Street, Loma Linda, CA 92350 USA

**Keywords:** Spirituality, Religion, Prayer, Pharmacy, Spiritual conversations

## Abstract

This exploratory study examined the spiritual care beliefs of California pharmacists and their perspectives and experiences with prayer and spiritual conversations with patients. An online survey was utilized to collect data from members of the California Society of Health-System Pharmacists in Spring 2021. Most of the 85 respondents (3.4% response rate) were Christian (61%), spiritual (73%) and had a spiritual or religious conversation with a patient (60%) and many had prayed for a patient (48%). Respondents believed that pharmacists can initiate prayer (32%) and spiritual conversations (45%) with a patient.

## Introduction

Most US Americans are religious and spiritual (Gallup Inc, 2021) and commonly express their spirituality or religiosity through prayer (Jors et al., 2015). National Gallup polls show that most Americans (75%) “sometimes” to “often” pray to God outside of religious services (Gallup Inc, 2021) and many pray during a time of crisis or illness (Gallup Inc, 2021; Jors et al., 2015). Many people pray for their health and the health of others (Gray et al., 2002; O'Connor et al., 2005; Twigg & Johnson, 2013), and one study reported that 90% of them believed that prayer improved their health (O'Connor et al., 2005). Prayer is also a source of comfort (Narayanasamy & Narayanasamy, 2008), hope, peace, coping and calmness to patients who believe in it (Ehman et al., 1999; French & Narayanasamy, 2011; Green, 2018; Kautz, 2008; Meraviglia, 2002; Mosley & Hill, 2003). Many patients believe that prayer is an important part of their health and treatment (Twigg & Johnson, 2013).

Many patients are open to praying with, and welcome offers of prayer from healthcare professionals (HCPs) in the clinical setting. A survey found that 57% of community pharmacy patients in Maryland were comfortable with their pharmacist praying with them (Twigg & Johnson, 2013). Furthermore, some patients ask pharmacists and pharmacy staff to pray for, or with them (Paul Gavaza et al., 2021a, 2021b; Purnell et al., 2019). A recent survey of 215 California pharmacists found that most were open to praying for, or with patients (P. Gavaza et al., 2021a, 2021b; Paul Gavaza et al., 2021a, 2021b), and many reported obliging to patient requests for prayer and spiritual conversations (Paul Gavaza et al., 2021a, 2021b; Purnell et al., 2019). These researchers also found that these pharmacists commonly used prayer to provide spiritual care (SC) to patients through privately praying for a patient (64%), offering to pray for a patient (29%), or encouraging a patient to pray (49%) (P. Gavaza et al., 2021a, 2021b). Pharmacists are encouraged to pray with and for receptive and interested patients (Rosenbaum, 2007).

Prayer is now recognized as an important complimentary/alternative therapy and an important part of whole person care (Harris et al., 1999; Hubbell et al., 2006; Watson, 2008). The nursing literature documents that some, but not all, patients want nurses to pray with them (French & Narayanasamy, 2011; Hubbartt et al., 2012; Rieg et al., 2006). Likewise, many patients also want their physicians to pray with them (King & Bushwick, 1994; MacLean et al., 2003; Siatkowski et al., 2008). Under the right conditions, praying with and for patients can be an important part of healthcare (Koenig et al., 1989). Likewise, some nurse authors have encouraged nurses to provide SC that includes prayer for patients who desire it in clinical practice (Hubbartt et al., 2012). Given this encouragement, it is not surprising that many nurses and physicians practicing in different clinical practice settings have reported praying for and with patients, or encouraging patients to pray (DeKoninck et al., 2016; Koenig et al., 1989; Taylor et al., 2018).

Furthermore, some patients also want to talk about spiritual aspects of their health and illness with HCPs during clinical encounters (Chatters, 2000; Levin, 1996; Matthews et al., 1998). Consequently, or perhaps because of internal prompts, nurses, physicians, and pharmacists have reported talking with patients about spiritual matters (Chatters, 2000; Daher et al., 2015; Levin, 1996; Matthews et al., 1998; Taylor et al., 2018). Many surveyed California pharmacists discussed spiritual issues with patients in practice (Paul Gavaza et al., 2021a, 2021b).

Although methods for measuring frequency of spiritual discourse differ in this literature—making comparisons difficult, it appears that fewer pharmacists are praying with and talking with patients about spiritual topics and issues than nurses (Balboni et al., 2011; DeKoninck et al., 2016; Koenig et al., 2017; Taylor et al., 2018). Only 34% of California pharmacists prayed with a patient at least “rarely”; 51%, however, talked to patients about a spiritual and/or religious topic at least “rarely,” despite having positive opinions about SC (P. Gavaza et al., 2021a, 2021b). This may be because some pharmacists believe that it is unprofessional, unethical, uncomfortable or none of their business to pray with patients or to have spiritual or religious (S/R) conversations with patients (Paul Gavaza et al., 2021a, 2021b). Some pharmacists may be unable to recognize a patients’ desire for prayer or spiritual conversation; some may not know how to provide spiritual or religious support. For some, this may be because they fear that their personal S/R beliefs and practices will become a barrier. Furthermore, many pharmacists believe that they should wait for patients to ask them first and thus are not proactively offering SC to patients due to fear of rejection, fear of offending patients, and an unwillingness to tread on patients’ privacy (Daher et al., 2015; P. Gavaza et al., 2021a, 2021b).

### Objectives

Given the paucity of evidence about how pharmacists interact with patients about their spiritual or religious concerns, the aim of this study was to explore pharmacist perspectives and experiences about prayer and spiritual conversations with patients. The specific objectives of the study are to describe from the self-reported perspectives of pharmacists what are their:

- Perspectives about prayer and S/R conversations in patient care
- Experiences with praying and having S/R conversation with patients, and
- Beliefs about spiritual care.
- Finally, the association between pharmacists’ demographic factors and their beliefs about spiritual care, perspectives, and experiences with prayer and spiritual conversations will be examined.

## Methods

This nonexperimental, cross-sectional survey study was approved by the Loma Linda University Health Institutional Review Board.

### Sample

Criteria for study participation included being employed as a pharmacist in a healthcare system. Participants were recruited from the membership of the California Society of Health-System Pharmacists (CSHP). At the time of the survey, CSHP had 3,500 members on its mailing list comprising pharmacists, retired pharmacists, pharmacy students, pharmacy technicians, pharmacy technician students, and associate members (Ryan Yamamura, California Society of Health-System Pharmacists, February 11th, 2021). This study only reports on findings from practicing pharmacists.

To recruit participants, the CSHP advertised and encouraged its members to complete the survey in multiple ways. Initially, the CSHP posted a brief description about the project as well as a link to the survey on its website in Spring 2021. In addition, CSHP also sent an e-mail to all members on the listserv in which the study was described, inviting members to complete the survey. Two weeks after the initial e-mail, a reminder e-mail was sent to all members on the listserv encouraging them to complete the survey if they had not done so already.

## Procedure

Data were collected using Qualtrics® during Spring 2021. When participants received the recruitment e-mail, a link to the survey was included. Clicking on the link took respondents to a consent page comprised of a cover letter that described the study objectives and provided the information requisite for consent. Potential respondents were asked to review and accept a consent statement prior to beginning the survey.

The survey was anonymous and did not collect any personally identifiable information about the respondents. The survey instrument comprised of 32 items and took about 5 min to complete. The study investigators wrote most of the items on the questionnaire.

The survey began with 12 Likert-type items which measured pharmacists’ beliefs about SC with response scales ranging from “strongly disagree” (1) to “strongly agree” (5). These items were borrowed from the “Oncology Nurse Spiritual Care Perspectives Survey” and adapted for use with pharmacists (Taylor et al., 1994). In addition, two questions from another nurse survey about spiritual care opinions were adapted for use to quantify the appropriateness of a pharmacist praying with a patient or conversing with a patient about spiritual or religious matters (Taylor et al., 2018). Response options ranged from “never” (1) to “the pharmacist can initiate regardless of circumstances” (7).

The survey also included four items to measure respondents’ past experience and future expectations regarding praying with patients and conversing with patients about spiritual or religious matters. Response options were yes/no. Examples of these items include: “Have you ever had a spiritual or religious conversation with a patient?” and “Would you ever pray for a patient in patient care?” Two additional items measured whether respondents perceived employer support for praying with patients and talking with patients about spiritual matters related to health. Response options included yes, no, and maybe.

The study also collected information about the respondents’ demographic, professional, and religious backgrounds. These variables included gender, ethnic/racial background, type of practice setting at primary place of employment, years practicing pharmacy, age, denominational affiliation, and whether or not they had received any training or education on spiritual care. Two items measured the respondents’ religiosity and spirituality: “How religious are you?” and “How spiritual are you?” with 5-point responses ranging from “not at all” (1) to “very” (5).

The last two items on the survey were open ended and gave respondents an opportunity to identify challenges or give comments. These items include: “What are some of the challenges that may impede pharmacists from praying for or with patients in patient care?” and “Please write here any comments you may have about pharmacists having prayer or health-related spiritual or religious conversations with patients.”

## Data Analysis

Data were exported directly from Qualtrics® to SPSS version 26 (IBM Corporation) for analysis. Descriptive statistics such as means, medians, standard deviations, and frequencies were computed for all applicable variables. We categorized data collected from the pharmacists’ responses to the items about the appropriateness of praying with and talking with patients about a spiritual topic as follows: a) the pharmacist can initiate regardless of circumstances (response option 1); b) the pharmacist can initiate depending on circumstances (response options 2 to 5); c) the pharmacist can initiate only after a patient raises the topic (response option 6); and d) never (response option 7). Analysis of variances (ANOVAs) were computed to compare the individual mean SC beliefs across the 12 items by these categories. A *p* value ≤ 0.05 was used as a criterion for significance for all statistical tests.

## Results

A total of 95 pharmacists responded to the survey; however, 10 incomplete responses were excluded from the analysis. Thus, results are based on 85 responses accounting for about 3.4% of the pharmacists on the mailing list. Most of the respondents were Christian (*n = *47, 61.0%), female (*n = *42, 52.5%), white/Caucasian (*n = *51, 62.2%), and had no training or education on SC (*n = *54, 69.2%). A majority self-reported that they were “somewhat” to “very” religious (*n = *51, 62.2%) and “somewhat” to “very” spiritual (*n = *60, 73.2%). Most did not work for a religiously affiliated institution (*n = *62, 75.6%), and on average had been practicing pharmacy for 28.3 years (SD = 17.2 years, range = 1–60 years). Nearly half (*n = *39, 47.6%) worked in a hospital setting (Table 1).

**Table 1:** Demographic and personal characteristics

Item	Frequency (%)
*Denominational affiliation (n = 77)*	
Christian (e.g., Protestant, Roman Catholic, Orthodox Christian)	47 (61.0%)
None/Non-traditional	6 (7.8%)
Buddhist	6 (7.8%)
Agnostic/Atheist/Humanist	8 (10.4%)
Jewish	5 (6.5%)
Other (e.g., Islam, Hindu, psychedelic sacrament-based religion)	5 (6.5%)
*Gender (n = 80)*	
Male	38 (47.5%)
Female	42 (52.5%)
*Ethnic/racial background (n = 82)*	
White	51 (62.2%)
Black or African American	2 (2.4%)
Asian	24 (29.3%)
Native Hawaiian or Pacific Islander	1 (1.2%)
Other	4 (4.9%)
*Type of practice setting at primary place of employment. (n = 82)*	
Community independent	5 (5.9%)
Community chain	3 (3.7%)
Hospital	39 (47.6%)
University	14 (17.1%)
Other	21 (25.6%)
*Do you work for a religiously affiliated institution? (n = 82)*	
Yes	18 (22.0%)
Maybe	2 (2.4%)
No	62 (75.6%)
*How religious are you? (n = 82)*	
Not at all	19 (23.2%)
A little	12 (14.6%)
Somewhat	24 (29.3%)
A lot	17 (20.7%)
Very	10 (12.2%)
*How spiritual are you? (n = 82)*	
Not at all	6 (7.3%)
A little	16 (19.5%)
Somewhat	23 (28.0%)
A lot	20 (24.4%)
Very	17 (20.7%)
*Training or education on spiritual care (n = 78)*	
Yes	14 (17.9%)
Maybe	10 (12.8%)
No	54 (69.2%)

## Beliefs About Spiritual Care

Table 2 presents the findings generated by the dozen items exploring beliefs related to SC. Particularly noteworthy are the results documenting how most respondents “strongly agreed” that “pharmacists should respect patients’ health-related religious and spiritual beliefs” (75.3%) and that “patients’ health issues are affected by spiritual/religious factors” (58.8%). While several other items garnered an agree/strongly agree response from roughly half the respondents, several items produced a more evenly distributed response pattern. Interestingly, the items related to what patients might want from a pharmacist in relation to S/R conversation or prayer received similar endorsement as the items about what is appropriate for a pharmacist to provide. These items were still skewed toward agreement (see Table 2).

**Table 2:** Respondents’ beliefs about spiritual care

Please rate your agreement with the following statements (*n = *85)	Mean (SD)	SD (1)	D (2)	Neutral (3)	A (4)	SA (5)
a) Some patients’ health issues are affected by spiritual/religious factors	4.45 (0.8)	1 (1.2)	2 (2.4)	5 (5.9)	27 (31.8)	50 (58.8)
b) Pharmacists should respect patients’ health-related religious and spiritual beliefs	4.68 (0.7)	1 (1.2)	–	3 (3.5)	17 (12.0)	64 (75.3)
c) Some patients desire spiritual or religious support from pharmacists. (*n = *84)	3.56 (1.0)	3 (3.6)	5 (6.0)	33 (39.3)	28 (33.3)	15 (17.9)
d) It is appropriate for pharmacists to have health-related spiritual and religious conversations with patients	3.61 (0.9)	2 (2.4)	8 (9.4)	24 (28.2)	38 (44.7)	13 (15.3)
e) It is appropriate for pharmacists to pray for patients who request it. (*n = *84)	3.68 (1.1)	4 (4.8)	8 (9.5)	23 (27.4)	25 (29.8)	24 (28.6)
f) It is important for pharmacists to pray for patients who request it. (*n = *84)	3.30 (1.2)	9 (10.7)	11 (13.1)	26 (31.0)	22 (26.2)	16 (19.0)
g) Patients are fine with pharmacists praying for them	3.38 (0.9)	3 (3.5)	5 (5.9)	43 (50.6)	25 (29.4)	9 (10.6)
h) Patients appreciate it when pharmacists pray for them	3.65 (1.0)	3 (3.5)	3 (3.5)	33 (38.8)	28 (32.9)	18 (21.2)
i) It is appropriate for a pharmacist to provide spiritual or religious support to patients who may need it	3.58 (1.1)	5 (5.9)	10 (11.8)	19 (22.4)	33 (38.8)	18 (21.2)
j) Pharmacists may object to serving a patient on account of their [pharmacist] spiritual or religious beliefs	2.82 (1.6)	31 (36.5)	8 (9.4)	5 (5.9)	27 (31.8)	14 (16.5)
k) It is sometimes helpful for a pharmacist to know patients’ spiritual or religious beliefs related to health	4.19 (0.9)	1 (1.2)	3 (3.5)	9 (10.6)	38 (44.7)	34 (40.0)
l) A patient’s spiritual concerns are none of the pharmacist’s business	2.45 (1.1)	19 (22.4)	26 (30.6)	28 (32.9)	7 (8.2)	5 (5.9)

1 = Strongly disagree (SA); 2 = Disagree (D); 3 = Neither agree nor disagree (Neutral); 4 = Agree (WA); 5 = strongly agree (SA)

## Experiences with Prayer and Spiritual Conversations with Patients

Although 53 (65.4%) respondents indicated that they would pray for a patient in patient care, 40 (48.2%) had prayed for a patient and 49 (59.8%) had a spiritual or religious conversation with a patient. Most respondents indicated that their companies/employers allowed or probably allowed them to pray for patients in patient care (*n = *72, 90.0%) and nearly all respondents (*n = *79, 96.3%) indicated that they would be or probably be willing to discuss spirituality if asked to do so by a patient (Table 3).

**Table 3:** Respondents’ experiences with praying and spiritual conversations with patients

Item	Frequency (%)
*Have you ever prayed for a patient? (n = 83)*	
Yes	40 (48.2%)
No	43 (51.8%)
*Would you ever pray for a patient in patient care? (n = 81)*	
Yes	53 (65.4%)
No	28 (34.6%)
*Have you ever had a spiritual or religious conversation with a patient? (n = 82)*	
Yes	49 (59.8%)
No	33 (40.2%)
*Would you be willing to discuss spirituality if asked to do so by a patient? (n = 82)*	
Yes	40 (48.8%)
Maybe	39 (47.6%)
No	3 (3.7%)
*Does your company/employer allow you to pray for patients in patient care? (n = 81)*	
Yes	21 (25.9%)
Maybe	53 (65.4%)
No	7 (8.6%)
*Does your company/employer allow you to talk to patients about spiritual matters that are related to their health? (n = 80)*	
Yes	32 (40.0%)
Maybe	40 (50.0%)
No	8 (10.0%)

## Appropriateness of Praying with and Conversing with Patients About a S/R Matter

A majority of respondents believed that it was appropriate for a pharmacist to pray with a patient if the patient initiated the request (*n = *51, 60.0%). Similarly, more than half (*n = *45, 52.9%) of the respondents believed that it was appropriate for a pharmacist to converse with a patient about S/R matters if the patient raised the topic. Very few viewed it appropriate to pray or initiate S/R conversation regardless of circumstances. Likewise, the converse was observed; very few responded that it would “never” be appropriate to pray (10.6%) or initiate S/R conversation (5.9%). Table 4 provides further details about respondents’ opinions regarding how context may shape their practice of prayer or S/R conversation with patients.

**Table 4:** Appropriateness of pharmacists praying with and conversing with a patient about a spiritual or religious matter

When is it appropriate for a pharmacist to pray with a patient in pharmacy practice? [Select all that applies] (*n = *85)	Frequency (%)
The pharmacist can initiate regardless of circumstances	2 (2.4)
The pharmacist can initiate if the pharmacist knows that prayer is important to the patient	27 (31.8)
The pharmacist can initiate if the pharmacist has a hunch that a prayer would be helpful	8 (9.4)
The pharmacist can initiate if the pharmacist has evidence indicating a prayer would be helpful	23 (27.1)
The pharmacist can initiate if the prayer has relevance to the patient’s health/disability	18 (21.2)
Only after a patient initiates a request for prayer	51 (60.0)
Never	9 (10.6)

When is it appropriate for a pharmacist to converse with a patient about spiritual or religious matters? [Select all that applies] (n = 85)	Frequency (%)
The pharmacist can initiate regardless of circumstances	5 (5.9)
The pharmacist can initiate if the pharmacist knows that spiritual or religious matters are important to the patient	38 (44.7)
The pharmacist can initiate if the pharmacist has a hunch that a spiritual or religious conversation would be helpful	18 (21.2)
The pharmacist can initiate if the pharmacist has evidence indicating a spiritual or religious conversation would be helpful	31 (36.5)
The pharmacist can initiate if a spiritual or religious conversation has relevance to the patient’s health/disability	28 (32.9)
Only after a patient raises the topic	45 (52.9)
Never	5 (5.9)

## Relationship Between Spiritual Care Beliefs and Spiritual Care Practice

After categorizing the responses to the two opinion items (related to when and if to initiate prayer or S/R conversation), ANOVAs were computed to determine if the means for individual spiritual belief items differed between these categories of opinion. A statistically significant difference in means for 11 of the 12 SC belief items was observed (Table 5). That is, with few exceptions, respondents who believed that pharmacists should talk with patients regardless of circumstances had significantly higher SC belief means than those who believed that pharmacists should talk only after patient raises topic. Likewise, the “only if” respondents had higher means for SC beliefs than the “never” respondents (Table 5).

**Table 5:** Relationship between R/S beliefs and categories of appropriateness for talking with patient about spiritual or religious matters

Item	Talk regard less	Talk depending	Talk only after patient ask	Never	F	*P*-value
a) Some patients’ health issues are affected by spiritual/religious factors	5.0 (0.0)	4.64 (0.5)	4.3 (0.8)	3.6 (1.7)	3.736	.014
b) Pharmacists should respect patients’ health-related religious and spiritual beliefs	4.8 (0.4)	4.86 (0.4)	4.6 (0.6)	3.8 (1.8)	4.098	.009
c) Some patients desire spiritual or religious support from pharmacists. (*n = *84)	4.4 (0.5)	3.7 (0.9)	3.4 (0.9)	2.4 (0.9)	4.657	.005
d) It is appropriate for pharmacists to have health-related spiritual and religious conversations with patients	5.0 (0.0)	4.1 (0.6)	3.2 (0.8)	2.6 (1.1)	15.500	< .001
e) It is appropriate for pharmacists to pray for patients who request it	4.6 (0.5)	4.3 (1.0)	3.3 (1.0)	2.6 (0.9)	10.109	< .001
f) It is important for pharmacists to pray for patients who request it	4.8 (0.4)	3.9 (1.1)	2.8 (1.1)	2.2 (1.1)	11.361	< .001
g) Patients are fine with pharmacists praying for them	4.0 (0.7)	3.9 (0.9)	3.0 *0.7)	2.6 (0.9)	10.346	< .001
h) Patients appreciate it when pharmacists pray for them	4.2 (0.8)	4.3 (0.8)	3.2 (0.8)	2.6 (0.9)	13.495	< .001
i) It is appropriate for a pharmacist to provide spiritual or religious support to patients who may need it	4.4 (0.9)	4.3 (0.8)	3.2 (1.0)	2.2 (1.1)	11.980	< .001
j) Pharmacists may object to serving a patient on account of their [pharmacist] spiritual or religious beliefs	3.0 (1.9)	3.29 (1.5)	2.4 (1.6)	3.0 (2.0)	1.698	.174
k) It is sometimes helpful for a pharmacist to know patients’ spiritual or religious beliefs related to health	4.8 (0.4)	4.4 (0.6)	4.2 (0.9)	2.8 (1.1)	6.954	< .001
l) A patient’s spiritual concerns are none of the pharmacist’s business	3.4 (1.1)	2.1 (1.0)	2.5 (1.0)	3.2 (1.8)	3.127	.030

With a few exceptions, this trend was similarly observed when comparing the SC belief means by opinions regarding when it was appropriate to initiate prayer with a patient. Only 7 of 12 SC belief items, however, showed a significant difference in means by these categories of opinion. See Table 6 for more details.

**Table 6:** Relationship between R/S beliefs and categories of appropriateness for praying with patients

Item	Pray depending	Pray only after patient ask	Never	F	*P*-value
a) Some patients’ health issues are affected by spiritual/religious factors	4.6 (0.5)	4.4 (0.8)	4.3 (1.3)	.894	.448
b) Pharmacists should respect patients’ health-related religious and spiritual beliefs	4.8 (0.4)	4.7 (0.6)	4.3 (1.4)	1.253	.296
c) Some patients desire spiritual or religious support from pharmacists	3.6 (0.9)	3.7 (0.8)	2.2 (0.8)	10.028	< .001
d) It is appropriate for pharmacists to have health-related spiritual and religious conversations with patients	4.0 (.7)	3.5 (0.9)	2.9 (0.9)	4.992	.003
e) It is appropriate for pharmacists to pray for patients who request it. (*n = *84)	4.5 (0.8)	3.5 (1.0)	2.2 (0.8)	14.371	< .001
f) It is important for pharmacists to pray for patients who request it. (*n = *84)	4.1 (0.9)	3.1 (1.1)	1.9 (0.9)	12.523	< .001
g) Patients are fine with pharmacists praying for them	4.0 (0.7)	3.3 (0.7)	2.3 (1.0)	11.439	< .001
h) Patients appreciate it when pharmacists pray for them	4.3 (0.7)	3.5 (0.7)	2.3 (1.0)	17.542	< .001
i) It is appropriate for a pharmacist to provide spiritual or religious support to patients who may need it	4.1 (0.9)	3.5 (1.0)	2.4 (1.3)	6.928	< .001
j) Pharmacists may object to serving a patient on account of their [pharmacist] spiritual or religious beliefs	3.2 (1.3)	2.6 (1.6)	3.0 (1.9)	.937	.427
k) It is sometimes helpful for a pharmacist to know patients’ spiritual or religious beliefs related to health	4.3 (0.6)	4.2 (0.8)	3.6 (1.3)	2.474	.068
l) A patient’s spiritual concerns are none of the pharmacist’s business	2.1 (0.9)	2.5 (1.1)	2.8 (1.5)	1.668	.181

## Discussion

The study results revealed that most respondents endorsed nearly all 12 SC items suggesting that overall this sample had positive beliefs about SC and generally acknowledged the role of spirituality in pharmacy practice. Most respondents believed that it is appropriate and important for pharmacists to address patients’ spirituality during patient care. Similarly, a previous study found that a majority of pharmacists agreed that they should practice in a spiritually sensitive manner (P. Gavaza et al., 2021a, 2021b). Other studies also found that HCPs acknowledge the role and importance of spirituality in healthcare (Koenig, 2004; McEwen, 2005; Taylor et al., 1994, 2018). These findings suggest that HCPs view spirituality as an important part of healthcare.

This study documented that most of the surveyed pharmacists had favorable beliefs about praying with patients. About three-fifths of the respondents agreed that it was appropriate for pharmacists to pray for patients who request it and about two-thirds indicated that they would pray with a patient. Previous studies also found that pharmacists prayed for and with patients (Daher et al., 2015; Paul Gavaza et al., 2021a, 2021b). Similarly, about two-thirds of physicians believed that praying with patients was an important part of healthcare (Balboni et al., 2011; Koenig et al., 1989, 2017). Nurses and physicians also pray for and with patients and encourage patients to pray in clinical practice (DeKoninck et al., 2016; Koenig et al., 1989, 2017; Taylor et al., 2018). Although most respondents were open to praying for and with patients, slightly less than half (48%) of the respondents had actually prayed for a patient. A similar proportion (47%) of nurses, physicians and physician assistants affiliated with a faith-based healthcare system also reported praying with patients (Koenig et al., 2017). Conversely, patients sometimes view prayer as valuable and an indicator of their HCP’s support and respect of their spiritual needs (Balboni et al., 2011; Paul Gavaza et al., 2021a, 2021b). Although it is encouraging that pharmacists are addressing patients’ spiritual needs through praying for patients, evidence suggests that pharmacists pray with patients less often than nurses (Balboni et al., 2011; DeKoninck et al., 2016; Koenig et al., 2017; Taylor et al., 2018). For example, about three-fifths of nurses in one study reported praying with patients (DeKoninck et al., 2016). Some respondents had never prayed for a patient despite having positive beliefs about praying with and for patients and most patients being open to praying with pharmacists (Twigg & Johnson, 2013). This may be explained by the many unique challenges that pharmacists face with respect to providing spiritual care as highlighted below. This is an area of opportunity for pharmacists interested in fostering holistic patient care. More training on how to respond to patients who need or request prayer may help more pharmacists to translate their positive opinions into practice.

We also found that nearly all the respondents (96%) were willing or probably willing to discuss spirituality if asked to do so by a patient and about three-fifths of the respondents agreed that it was appropriate for pharmacists to have health-related spiritual and religious conversations with patients. However, about two-thirds of respondents had a spiritual or religious conversation with a patient. Nurses and physicians also discuss spiritual topics and issues with patients in the clinical setting (Chatters, 2000; Levin, 1996; Matthews et al., 1998; Taylor et al., 2018). In addition, patients are open to talk to physicians and pharmacists about spiritual aspects related to their health and illness (Chatters, 2000; Levin, 1996; Matthews et al., 1998; Twigg & Johnson, 2013).

Only 9% and 10% of the respondents indicated that their employers/company did not allow them to pray for patients in patient care and talk to patients about spiritual matters that are related to their health, respectively. Many respondents (50% and 65%) responded “maybe” to both items suggesting that the respondents were unclear on their companies/employers’ policies in this regard. Most of the respondents were employed in the acute care/hospital setting and thus could be expected to be more receptive to the role of SC given the Joint Commission’s recognition of the importance of SC in healthcare institutions. Pharmacists practicing predominantly in community pharmacies report that most employers/companies do not allow pharmacists to pray or talk to patients about spiritual or religious issues (P. Gavaza et al., 2021a, 2021b).

About two-thirds of the respondents indicated that it was appropriate for a pharmacist to pray with a patient only after a patient initiates a request for prayer. Despite the fact that many patients welcome HCPs’ (including pharmacists’) offers of prayer (Twigg & Johnson, 2013), many pharmacists prefer to wait for patients to ask them first as reported elsewhere (Daher et al., 2015; Paul Gavaza et al., 2021a, 2021b). Similar findings have been reported among nurses (Millison & Dudley, 1992; Taylor et al., 1994). A greater proportion of nurses (12%) indicated that a nurse can initiate prayer with patients regardless of circumstances (Taylor et al., 2018). Our sample seems to be more conservative or cautious on this subject than nurses. It is possible that some pharmacists may not initiate prayer and spiritual conversations with patients who need and would benefit from them fearing that doing so may be interpreted by patients as proselytizing.

More training should be provided to pharmacists to help them better navigate the delicate task of addressing patients’ spiritual needs. Furthermore, there is need for policy guidance on how and when to provide SC in pharmacy practice including praying with or having a spiritual conversation with patients. Pharmacists who know a patient’s spiritual background and have an established relationship with the patient in need of prayer can appropriately, respectfully, and ethically address patients’ spiritual needs through SC.

### Limitations of the Study

The findings of this exploratory study should be interpreted in light of several limitations. First, the analysis included only 85 responses, representing a response rate of 3.4%. A higher response rate might have been achieved if financial incentives had been offered to potential participants. Second, the study relied on a convenience sample, likely comprising individuals particularly interested in the study's topic. Consequently, the results may reflect a bias toward favorable attitudes regarding prayer and spiritual conversations, leading to predominantly positive findings. Furthermore, the respondents were drawn from a single pharmacy organization in one state, limiting the generalizability of the results to all pharmacists within the state or across the USA. Thus, the reported rates likely represent a best-case scenario among those interested in spiritual practices.

Another limitation is the absence of psychometric validation for the survey items used in the study. While the original nurse version of the SC beliefs items demonstrated content validity and test–retest reliability, it is unclear how these items perform in a pharmacist population. Additionally, small sample sizes for some opinion categories may have weakened the statistical power of the ANOVAs reported in Tables 5 and 6, potentially affecting the robustness of the findings.

## Conclusion

Most pharmacists surveyed believed that spirituality and SC including prayer and spiritual conversations with patients were important parts of patient care. Many respondents prayed and had spiritual conversations in patient care. Further research on SC and how to integrate it within holistic pharmacy care is warranted.
